# Persistence of non-typhoidal *Salmonella* across hosts and environments: a One Health perspective

**DOI:** 10.3389/fmicb.2026.1830232

**Published:** 2026-07-21

**Authors:** Amer Abdelgany, Alex Wong, Jiewen Guan

**Affiliations:** 1 Department of Biology, Carleton University, Ottawa, ON, Canada; 2 Ottawa Laboratory (Fallowfield) – Animal Health Research and Development, Canadian Food Inspection Agency (CFIA), Ottawa, ON, Canada

**Keywords:** antimicrobial resistance, environmental persistence, host adaptation, non-typhoidal *Salmonella*, One Health

## Abstract

*Salmonella* (*S.*) *enterica* remains a major global pathogen, with non-typhoidal *Salmonella* (NTS) causing millions of infections in humans and animals each year. While acute salmonellosis is well characterized, less is understood about the factors that enable NTS to persist across hosts, food systems, and environmental reservoirs. In this review, we integrate epidemiological, experimental, and genomic evidence on bacterial, host, and environmental determinants that promote long-term survival and persistence within a One Health framework. Persistence is examined across human, animal, and environmental contexts, encompassing prolonged host carriage, endemic circulation in animal reservoirs, and long-term survival in agro-environmental and food-processing settings. Within hosts, biofilm formation, intracellular adaptation, and stress-response pathways contribute to chronic colonization, while persistence in soil, water, manure, and on-farm or food-processing environments supports environmental endurance and ongoing transmission. Persistence strategies vary among NTS lineages and are shaped by ecological context and antimicrobial pressure, underscoring the need for integrated approaches to surveillance and control. Addressing these interconnected challenges requires a One Health perspective that links biological mechanisms with environmental and genomic surveillance–guided interventions across clinical, agricultural, and environmental sectors to disrupt persistence and transmission cycles and reduce the global public health burden of *Salmonella* infections.

## 1 Introduction

*Salmonella* is a rod-shaped, Gram-negative bacterium causing a wide range of gastrointestinal and systemic illnesses. It is one of the leading foodborne pathogens globally; it is responsible for an estimated 93.8 million cases of salmonellosis and approximately 155,000 deaths annually (Galán-Relaño et al., 2023). Most cases are sporadic and not part of recognized outbreaks (Popa and Papa, 2021). Salmonellosis imposes a substantial health burden in both low- and high-income countries, and it incurs high economic costs in terms of healthcare and productivity losses (Majowicz et al., 2010). *Salmonella* is commonly found in poultry and livestock, including swine and cattle, which serve as primary reservoirs (Shaji et al., 2023). *Salmonella* colonizes the intestinal tract but may also be present on skin and feathers, especially in poultry. Humans can acquire *Salmonella* infections through the handling of infected animals or meat, consuming undercooked meat, or by contact with contaminated water, leading to clinical illness (Kabir, 2010).

*Salmonella enterica* is classified into six subspecies based on genomic and biochemical characteristics: enterica (I), salamae (II), arizonae (IIIa), diarizonae (IIIb), houtenae (IV), and indica (VI) (Porwollik et al., 2004). *S. enterica* subspecies enterica (I) is the most clinically relevant, accounting for nearly 99% of infections in warm-blooded animals and humans (Ayuti et al., 2024). Beyond subspecies, the Kauffmann–White classification divides *Salmonella* into over 2,400 serotypes based on two key antigenic markers: the somatic (O) antigens, which are components of the lipopolysaccharide layer on the bacterial surface, and the flagellar (H) antigens, which are associated with the bacterial flagella and may occur in one or two phases (Wang et al., 2020).

Moreover, *Salmonella enterica* subspecies enterica is classified into two major groups: typhoidal *Salmonella* (TS) and non-typhoidal *Salmonella* (NTS). Typhoidal serovars (such as *S.* Typhi) cause systemic illnesses such as typhoid fever, which remains a serious public health concern. According to the World Health Organization, typhoid fever affects approximately 9–20 million people per year, resulting in an estimated 110,000 deaths worldwide (World Health Organization [WHO], 2023). It is particularly prevalent in South Asian countries, such as India, Bangladesh, and Pakistan, as well as in parts of sub-Saharan Africa (Hancuh, 2023). TS is more commonly found in lower- and middle-income regions than in high-income countries (Gal-Mor et al., 2014).

Non-typhoidal *Salmonella* are *Salmonella enterica* serovars that typically cause self-limited gastroenteritis in humans. Nonetheless, infection can be serious in young children, the elderly, and immunocompromised individuals. Invasive non-typhoidal *Salmonella* (iNTS) disease has emerged as one of the main causes of life-threatening bacteremia in regions with high HIV prevalence and other immunocompromising factors. Some NTS serovars, including *S.* Enteritidis, *S.* Typhimurium, *S.* Heidelberg, and *S.* Newport, have a broad host range (Cheng et al., 2019). These pathogens are often harbored by poultry, cattle, and other animals, which rank among the leading causes of foodborne salmonellosis worldwide (Lamichhane et al., 2024). Certain *Salmonella* lineages have evolved host adaptation, thriving primarily in one animal species while rarely infecting others. Such host-restricted strains frequently cause invasive disease within their reservoir hosts and can show severe extra-intestinal infections when transmitted to humans (Soliani et al., 2023).

In addition to its ability to colonize animals and humans, *Salmonella* also demonstrates remarkable environmental persistence, enabling it to survive in harsh conditions. NTS has been reported to survive for up to 5 months in diverse soil environments (Alegbeleye and Sant’Ana, 2023). Together, evidence shows that *Salmonella* circulates among humans, animals, and environmental reservoirs, with transmission across these three compartments being well documented (Wilson et al., 2025). This connected distribution underscores the need for coordinated, multi-sectoral strategies to limit the spread and persistence of *Salmonella*.

Adopting a One Health approach is therefore essential. One Health recognizes that human, animal, and environmental health are interdependent and that effective control of *Salmonella* requires integrated surveillance, multidisciplinary research, and collaborative interventions across these areas. In this context, by studying how biological, ecological, and host-related factors interact, One Health provides a comprehensive framework to understand the pathways that support *Salmonella* persistence.

The concept of “persistence” is well established in the literature. In this review, the term is used to describe the capacity of NTS to maintain viable populations over extended time scales despite host immune pressure, antimicrobial exposure, or adverse environmental conditions. In human hosts, persistence encompasses prolonged or intermittent fecal shedding and asymptomatic carriage following apparent clinical recovery, rather than the duration of acute disease alone. Similarly, in animal reservoirs, persistence refers to long- term colonization and endemic circulation within herds or flocks, often in the absence of overt clinical signs. In environmental contexts, persistence refers to *Salmonella*’s ability to survive and remain transmissible in soil, water, manure, food- processing environments, and farm infrastructure for months to years. Collectively, these overlapping manifestations of persistence sustain transmission cycles across human, animal, and environmental compartments, forming the basis of a One Health persistence framework.

**FIGURE 1:**
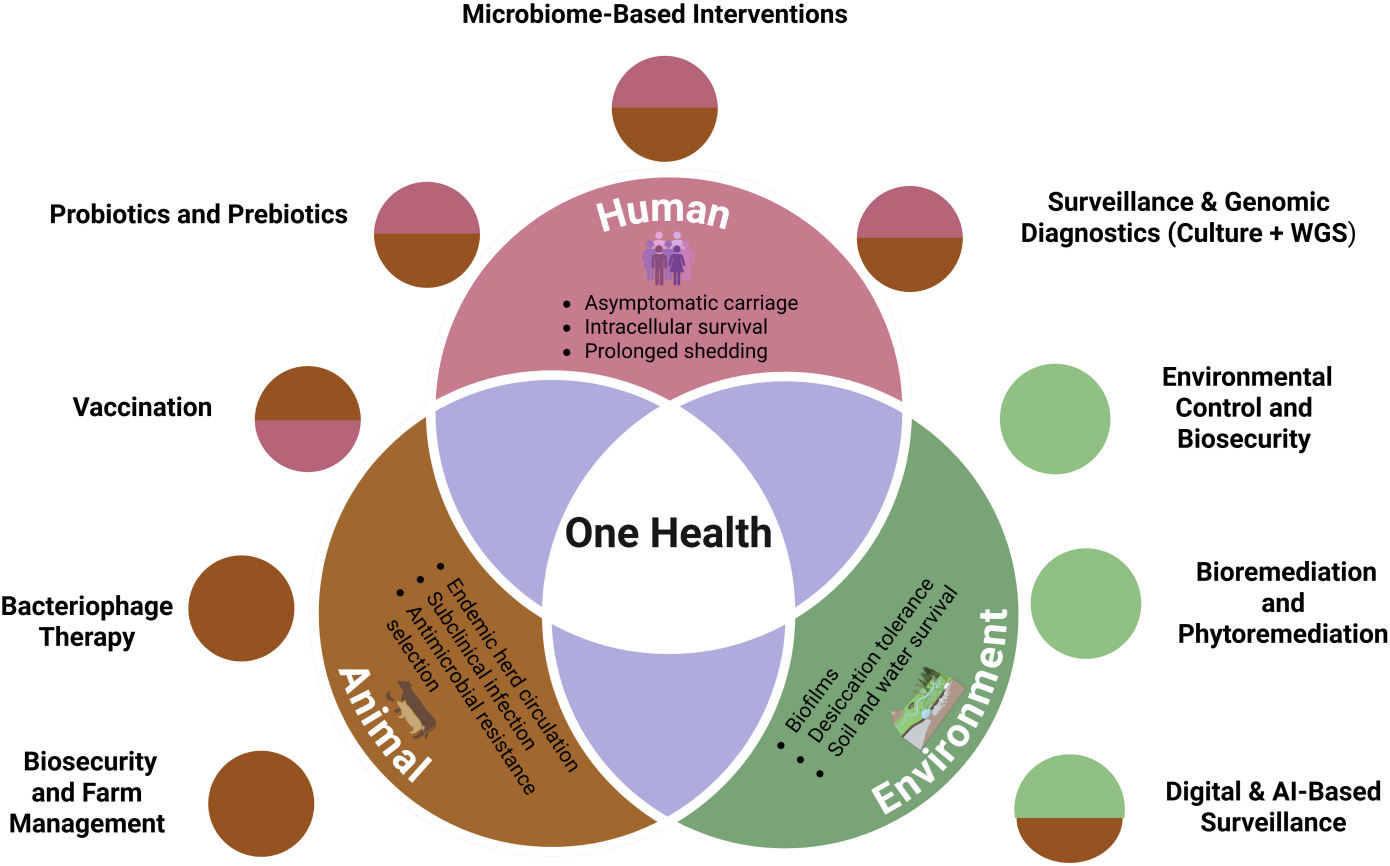
One Health framework for persistence and mitigation of non-typhoidal *Salmonella*. Non-typhoidal *Salmonella* persists through interconnected human, animal, and environmental reservoirs via mechanisms including asymptomatic carriage, intracellular survival, prolonged shedding, biofilm formation, desiccation tolerance, soil and water survival, and antimicrobial resistance selection. These persistence mechanisms enable sustained circulation and repeated re-introduction across the human–animal–environment interface, highlighting the need for coordinated biological, environmental, and surveillance-based interventions within a One Health framework. Generated using BioRender.com.

In this review, we focus on NTS, with particular emphasis on the factors that enable its persistence at the human–animal–environment interface. From a One Health perspective, understanding these interactions is critical for developing effective mitigation strategies and reducing the overall public health burden of *Salmonella*. We outline key strategies to combat NTS, including integrated surveillance and targeted interventions across animal, human, and environmental sectors to interrupt transmission and reduce persistence. To provide a conceptual overview, Figure 1 illustrates the persistence of NTS across human, animal, and environmental reservoirs within a One Health framework, together with the key intervention points that can disrupt these persistence cycles.

## 2 Literature search strategy

Literature for this narrative review was identified through iterative, topic- driven, and section- specific searches of PubMed and Google Scholar. Search terms were selected according to the thematic focus of each section, including mechanisms of persistence, host- associated carriage, environmental survival, epidemiology, antimicrobial resistance, monophasic strains, and mitigation strategies.

Sections addressing mechanistic aspects of persistence prioritized experimental and laboratory- based studies, whereas sections focused on persistence dynamics and epidemiology emphasized recent field, surveillance, and population- based studies. Recent review articles were used to frame each major topic, with primary research papers selected to illustrate key mechanisms or representative examples. Only peer- reviewed articles published in English were included; conference abstracts and non-peer-reviewed sources were excluded. Given the interdisciplinary scope of the subject, this review adopts a narrative synthesis approach rather than a formal systematic review, integrating evidence across human, animal, and environmental domains within a One Health framework.

## 3 Persistence in humans

Persistence of NTS in humans extends beyond acute gastroenteritis, reflecting the pathogen’s ability to evade immune clearance and establish chronic carriage. Following infection, *Salmonella* can survive intracellularly within macrophages and epithelial cells, enabling chronic shedding even after clinical recovery (Marzel et al., 2016; Monack et al., 2004).

Non-typhoidal *Salmonella* infections can lead to a carrier state characterized by persistent intestinal colonization and prolonged fecal shedding in both symptomatic and asymptomatic individuals. Shedding typically lasts several weeks, with a median duration of approximately 5 weeks, extending to around 7 weeks in children under 5 years of age (Rohringer et al., 2025). Although less common, chronic carriage (>1 year) occurs in a small proportion of cases, particularly in young children (∼2.6%) compared to <1% in the general population (Rohringer et al., 2025). Persistent carriers often exhibit intermittent mild diarrhea (in ∼65% of cases), suggesting subclinical inflammatory activity (Marzel et al., 2016; Waldner et al., 2012).

At the mechanistic level, *Salmonella* long-term survival involves adaptation to hostile host niches such as the gallbladder, lymphatic tissues, and intestinal mucosa, where biofilm formation and macrophage survival protect bacteria from immune clearance (Lawley et al., 2006). These processes also confer antimicrobial tolerance, particularly in multidrug-resistant strains (Tack et al., 2020). Host factors such as immune suppression (e.g., HIV infection, diabetes, chemotherapy) and antibiotic-induced dysbiosis further extend carriage duration by disrupting colonization resistance (Zhou et al., 2026; Rivera-Chávez et al., 2016).

Human persistence thus reflects a spectrum from transient shedding to long-term carriage driven by pathogen adaptation, host susceptibility, and antimicrobial exposure. This prolonged carriage reinforces environmental and zoonotic transmission, linking the clinical and ecological dimensions of NTS. Importantly, prolonged human carriage and intermittent shedding must be considered in the context of upstream reservoirs, as antimicrobial use and selection in livestock (Section “6 Animal reservoirs and host range”) and continued environmental exposure through contaminated water and food systems (Section “7 Environmental persistence and transmission routes”) can repeatedly re-seed human infections and sustain persistence across the One Health continuum.

## 4 Global epidemiology and burden

Non-typhoidal *Salmonella* remains a leading cause of bacterial foodborne disease worldwide (Galán-Relaño et al., 2023). It is estimated that tens of millions of enteric infections occur annually, most linked to animal-derived foods such as poultry, eggs, beef, and pork. Despite decades of control programs, *Salmonella* continues to impose a major global public-health and economic burden, responsible for billions of dollars in medical costs and productivity losses each year (European Food Safety Authority [EFSA], and European Centre for Disease Prevention and Control [ECDC], 2022, 2024). This burden is increasingly compounded by the global emergence and spread of antimicrobial-resistant non-typhoidal *Salmonella*, which complicates treatment outcomes, contributes to prolonged infection and carriage, and sustains transmission across human, animal, and environmental reservoirs (Tack et al., 2020; Kumar et al., 2025).

### 4.1 Global and regional trends

Recent Global Burden of Disease (GBD) analyses estimate that the worldwide incidence of NTS increased at an average annual rate of 0.45% between 1990 and 2021 (Shi and You, 2025). Surveillance data indicated that *Salmonella* infections were stable or slightly declining in most high-income countries but remained high in many developing regions. In the European Union, *Salmonella* cases dropped from around 96,000 in 2010 to 60,000 in 2019, which was driven by poultry-flock vaccination and improved hygiene (European Food Safety Authority [EFSA], and European Centre for Disease Prevention and Control [ECDC], 2022). In the United States, the CDC’s FoodNet system reported approximately 17.5 cases per 100,000 population in 2019, similar to the previous decade (Centers for Disease Control and Prevention [CDC], 2024).

By contrast, in parts of Southeast Asia and sub-Saharan Africa, people still experience much higher endemic levels. *S.* Typhimurium ST313 and *S.* Enteritidis cause up to 3 million invasive cases annually, with a mortality rate between 14% and 25% (Van Puyvelde et al., 2019; Preciado-Llanes et al., 2020). In parts of Central Africa, *Salmonella* is considered the main cause of enteric illness, accounting for over 50% of acute diarrhea cases reported between 1998 and 2022 (Zong Minko et al., 2024). These differences highlight the gap between resourced and resource-limited settings and persistent regional inequalities in food safety and sanitation, particularly where access to clean water and veterinary oversight is limited.

### 4.2 Impact of the COVID-19 pandemic

Pandemic- related restrictions led to a marked but short- term decrease in reported NTS incidence. In 2020, both CDC FoodNet and the EU One Health Zoonoses platform reported a 25%–35% reduction in laboratory- confirmed salmonellosis compared to 2019 (European Food Safety Authority [EFSA], and European Centre for Disease Prevention and Control [ECDC], 2022; Centers for Disease Control and Prevention [CDC], 2024). This reduction is likely attributable to decreased travel, limited restaurant dining, and reduced international food trade, rather than an actual decline in pathogen prevalence across environmental and animal reservoirs. As restrictions were lifted in 2021–2022, reported NTS cases rebounded. In the EU, notifications increased to 65,208 cases in 2022, while U.S. incidence returned to approximately 18 per 100,000 (Centers for Disease Control and Prevention [CDC], 2024). In Canada, national surveillance recorded a nadir of 3,360 laboratory- confirmed cases in 2021 (8.8 per 100,000), followed by increases in 2022 and 2023 approaching pre- pandemic levels (Public Health Agency of Canada [PHAC], 2024, 2025a). This rapid resurgence supports the conclusion that pandemic control measures temporarily disrupted exposure and detection, rather than interrupting the underlying persistence of NTS within food systems and environmental reservoirs. Notably, outbreaks reported in early 2025 involving multidrug- resistant serovars further indicate that long- term persistence in production and processing environments continued despite short- term behavioral changes.

Overall, the COVID- 19 pandemic had a heterogeneous impact on *Salmonella* surveillance, with most studies reporting decreased case notifications compared with pre- pandemic periods (Mughini-Gras et al., 2021; Wang T. et al., 2025). This pattern is consistent with reduced fecal–oral transmission opportunities during lockdowns. However, reduced healthcare- seeking behavior, laboratory capacity constraints, and temporary surveillance disruptions likely contributed to under- ascertainment of milder infections, complicating interpretation of observed declines (Emdin et al., 2025). Importantly, several studies reported contrasting trends. Surveillance data from the Netherlands showed decreased overall incidence but increased invasive infections among elderly populations, whereas data from China indicated higher hospitalization rates during the pandemic, particularly among young children (Mughini-Gras et al., 2021; Wang T. et al., 2025). These findings suggest that vulnerable populations and invasive disease manifestations may be less influenced by changes in exposure behavior and more closely linked to host susceptibility and persistent sources of infection.

COVID-19-associated non-pharmaceutical interventions, such as early detection screening, quarantine measures, and movement restrictions, were linked to declines in many enteric bacterial infections; however, NTS incidence increased in some settings (Huang et al., 2024). Genomic analyses further indicated shifts in antimicrobial resistance, virulence, and disinfection tolerance, underscoring the complex interaction between environmental persistence, selective pressure, and transmission during public- health disruptions (Huang et al., 2024). The pandemic also altered antimicrobial usage patterns. Despite low rates of bacterial co- infection in COVID- 19 patients (∼8%), empirical antibiotic use reached up to 72% in some settings (Rawson et al., 2020). Such widespread antimicrobial exposure may indirectly promote NTS persistence by disrupting gut microbiota and selecting for resistant or stress- tolerant strains, although outcomes varied across regions (Emdin et al., 2025). Short- term studies limited to 2020 often detected minimal resistance changes, whereas data from 2021 to 2022 demonstrated increasing resistance following rebound antimicrobial prescribing, indicating delayed effects (Emdin et al., 2025). In low- and middle- income countries, where self- medication is more common, antimicrobial- resistant bacteria were more frequently detected following COVID-19-associated antibiotic use (Khanam et al., 2025).

Collectively, these observations indicate that the COVID- 19 pandemic did not fundamentally alter the capacity of NTS to persist, but instead modulated exposure, detection, and selective pressures acting on existing reservoirs. Disruptions to surveillance, gut- microbiome perturbation, and increased antimicrobial use may, in some contexts, have favored prolonged persistence and adaptive evolution of resistant NTS lineages. These adapted populations can subsequently be shed and reintroduced into human, animal, and environmental reservoirs, reinforcing the need for resilient One Health- oriented surveillance and control systems capable of maintaining continuity during public- health disruptions.

### 4.3 Serovar distribution and environmental drivers

Serovar distribution varies by region. In Canada, *S.* Enteritidis, *S.* Typhimurium, and *S.* Heidelberg are among the most prevalent serovars. In the United States, *S.* Heidelberg has been linked to several outbreaks associated with poultry and ready-to-eat foods, along with *S.* Infantis and *S.* Kentucky ST198 (European Centre for Disease Prevention and Control [ECDC], 2024; European Food Safety Authority [EFSA], and European Centre for Disease Prevention and Control [ECDC], 2022). In Thailand, *S.* Enteritidis, *S.* Stanley, and *S.* Weltevreden are common, while in Tunisia, *S.* Enteritidis, *S.* Typhimurium, and *S.* Amsterdam are dominant (Cheng et al., 2019). These differences likely reflect the effects of local livestock production, dietary practices, and trade patterns.

Seasonal and environmental factors also affect serovar distribution, with temperature emerging as an important driver of NTS persistence under ongoing climate warming trends. NTS incidence consistently increases during spring and summer, when ambient temperatures above 25 °C enhance bacterial growth and survival in food, water, and environmental reservoirs (Centers for Disease Control and Prevention [CDC], 2024). Rising global temperatures may therefore prolong the seasonal transmission window of NTS and shift peak incidence earlier in the year, particularly in temperate regions.

Climate change–associated extreme weather events, including flooding and heavy precipitation, can mobilize *Salmonella* from manure- amended soils into surface waters, promoting environmental persistence at the interface between farms, wildlife, and human populations (European Food Safety Authority [EFSA], and European Centre for Disease Prevention and Control [ECDC], 2022). These events may also increase contamination pressure outside traditional seasonal peaks, reducing the predictability of NTS transmission dynamics. Climatic conditions, together with international trade and travel, therefore contribute to sustained year- round transmission. Although temporary declines in reported incidence occurred during the COVID- 19 pandemic, *Salmonella* prevalence rapidly returned to pre- pandemic levels, highlighting the resilience of NTS transmission cycles under changing environmental conditions. Collectively, these observations emphasize the need for coordinated, climate- informed surveillance across agricultural, environmental, and clinical sectors.

## 5 Clinical manifestations of NTS

Non-typhoidal *Salmonella* infections in humans show a broad range of clinical symptoms from mild, self-limiting gastroenteritis to invasive, life-threatening diseases. Gastroenteritis is the most frequent presentation, typically developing 6–72 h after ingestion of contaminated food or water. Patients experience diarrhea, abdominal pain, fever, nausea, and vomiting that generally resolve within 4–7 days (World Health Organization [WHO], 2018; Centers for Disease Control and Prevention [CDC], 2024). While most healthy adults recover without antimicrobial therapy, young children, elderly individuals, pregnant women, and immunocompromised hosts such as those with HIV infection or undergoing chemotherapy are at increased risk for severe or prolonged illness (Gal-Mor et al., 2014).

Invasive non-typhoidal *Salmonella* disease occurs when bacteria break through the intestinal barrier and enter the bloodstream, producing bacteremia, meningitis, or focal infections such as osteomyelitis (Tack et al., 2020). iNTS is particularly prevalent in regions with high HIV and malaria co-infection rates or malnutrition, where fatality ratios can reach between 14% and 25% (Van Puyvelde et al., 2019; Preciado-Llanes et al., 2020). Most of these invasive isolates belong to *S.* Typhimurium and *S*. Enteritidis, which display host-adaptation traits that facilitate systemic spread.

Following recovery, a subset of patients continues to shed NTS for weeks or months, acting as chronic or intermittent carriers. Marzel et al. (2016) reported recurrent infections caused by genetically identical isolates, confirming long-term intestinal persistence within the host. Post-infectious symptoms such as reactive arthritis and irritable bowel syndrome have also been documented (Hannu et al., 2002; Klem et al., 2017).

Antimicrobial therapy is generally reserved for severe or invasive cases, yet rising resistance to fluoroquinolones, third-generation cephalosporins, and ampicillin complicates management (Tack et al., 2020). Prolonged shedding of multidrug-resistant serovars increases the likelihood of household and community transmission, such that persistence in humans is not limited to the duration of infection but extends to chronic carriage and environmental dissemination. Regional variation in severity and persistence reflects differences in exposure and antimicrobial use patterns across populations, emphasizing the importance of understanding global epidemiological trends in non-typhoidal *Salmonella*.

## 6 Animal reservoirs and host range

Non-typhoidal *Salmonella* serovars exhibit a broad spectrum of host specificity, ranging from highly generalist serovars to those adapted or restricted to a particular animal host (Soliani et al., 2023). Broad host-range serovars (host-generalists) such as *Salmonella enterica* serovars Typhimurium and Enteritidis are capable of infecting a wide variety of mammals, birds, reptiles, and other animals and are among the most prevalent causes of human salmonellosis worldwide (Muslin et al., 2025). In contrast, narrow host-range (host-restricted) NTS serovars, characterized by adaptation to a single primary host species, with increased virulence and more severe disease in that reservoir and only sporadic infections in other species (Soliani et al., 2023). Examples include *S.* Dublin in cattle (Campioni et al., 2022), *S.* Choleraesuis in swine (Soliani et al., 2023), and *S.* Gallinarum in poultry (Eswarappa et al., 2009), each largely associated with a specific animal host. These differences in host range are pivotal to *Salmonella* persistence; host-generalists can circulate across diverse ecological niches, whereas host-specific serovars establish enduring endemic cycles in their favored animal populations, with both scenarios contributing to a stable zoonotic reservoir (Soliani et al., 2023; Velasquez-Munoz et al., 2024).

### 6.1 Domestic poultry

Domestic poultry are a key global reservoir of NTS, serving as hosts for broad host-range serovars such as *S.* Enteritidis and *S.* Typhimurium, which colonize the intestinal tract of adult birds with minimal symptoms, but frequently contaminate meat and eggs (Shaji et al., 2023). They often cause asymptomatic or subclinical infection in their animal hosts, enabling prolonged fecal shedding and environmental contamination without drawing attention to the infection (Davies et al., 2019; Sreekantapuram et al., 2021). For example, adult poultry infected with *S.* Enteritidis or *S.* Typhimurium may show little illness manifestation, even as they shed bacteria into barns or food products. This silent carriage facilitates long-term colonization in farms and along the food chain. *S.* Enteritidis is particularly capable of invading the hen’s reproductive tract and causing internal egg contamination, thereby contributing to many human salmonellosis cases (Wigley, 2024b). In this context, young chicks exposed to these serovars may develop clinical disease, whereas older birds tend to become asymptomatic carriers that shed bacteria (Shaji et al., 2023).

By contrast, poultry-adapted serovars, such as *S.* Pullorum and *S.* Gallinarum, cause pullorum disease and fowl typhoid, respectively, systemic illnesses with high mortality in young birds and vertical transmission via eggs (Farhat et al., 2024; Shen et al., 2022).

*Salmonella* Gallinarum in chickens often causes systemic, sometimes fatal infections in their primary hosts (Sreekantapuram et al., 2021; Velasquez-Munoz et al., 2024). Surviving animals can become chronic carriers – for example, chickens that recover from *S.* Gallinarum can harbor the bacteria for life (Sreekantapuram et al., 2021). These carrier states enable the pathogen to persist in a herd or flock for years (Eriksson et al., 2018; Velasquez-Munoz et al., 2024).

Successful elimination of these poultry-adapted serovars from commercial stock has required rigorous testing and culling, for example, in Canada, the United States, Australia, and most European countries (Kang et al., 2024). However, in regions where fowl typhoid or pullorum disease remains endemic, these serovars persist in backyard flocks or wild birds (Eriksson et al., 2018). Importantly, poultry-adapted serovars rarely infect humans; instead, the broad host-range serovars originating from poultry continue to pose a serious zoonotic risk (Wigley, 2024b).

### 6.2 Cattle

Cattle are an important reservoir of NTS, serving as hosts for a wide range of serovars with varying host specificity. Among these, common broad-host-range serovars such as *S.* Typhimurium, *S.* Newport, and *S.* Enteritidis frequently infect cattle but are not cattle-specific (Kovac et al., 2017). These generalist serovars often cause enterocolitis in young calves, while adult cattle may shed them asymptomatically, contaminating beef, raw milk, and farm environments (Cummings et al., 2009; Holschbach and Peek, 2018). Consequently, outbreaks of human salmonellosis have been linked to these serovars; for example, in the United States, *S.* Newport has been associated with beef products and *S.* Typhimurium with raw milk, with many isolates harboring multiple antimicrobial resistance genes (Cummings et al., 2009; Holschbach and Peek, 2018).

By contrast, the most significant cattle-adapted serovar is *S.* Dublin, which causes severe systemic disease with fever, pneumonia, diarrhea, and abortion in cows (Nielsen, 2013). *S.* Dublin is particularly difficult to eradicate, as recovered animals may become long-term carriers, shedding bacteria in feces or milk and thereby enabling persistence and spread within herds (Holschbach and Peek, 2018; Nielsen, 2013). Surviving cattle can become chronic carriers; for example, cattle infected with *S.* Dublin may develop latent infections (e.g., in the gallbladder or lymphatic tissues) and intermittently shed the organism. These carrier states enable the pathogen to persist in a herd or flock for years (Velasquez-Munoz et al., 2024).

Importantly, *S.* Dublin is also zoonotic: although less common in humans, infections are often invasive, frequently causing bacteremia, and many isolates exhibit multidrug resistance (Velasquez-Munoz et al., 2024; Sekhwal et al., 2025). Beyond this dominant serovar, other serovars such as *S.* Cerro have emerged in dairy cattle, typically causing subclinical infection and to date rarely affecting humans (Cummings et al., 2010; Cohn et al., 2022).

### 6.3 Swine

Swine are a major reservoir of NTS, carrying both broad-host-range and pig-adapted serovars. Generalist serovars such as *S.* Typhimurium are most prevalent in herds. *S.* Typhimurium often causes diarrhea and fever in young pigs but is frequently shed without signs in finishing pigs, contaminating pork at slaughter. In recent years, the emergence of monophasic variants of *S.* Typhimurium (particularly *S.* 4,[5],12:i:-) has further amplified the role of swine in NTS persistence, as these strains are strongly associated with pig production systems, multidrug resistance, and efficient transmission within herds (Mastrorilli et al., 2018). *S.* Derby is considered swine-adapted due to its high prevalence in pigs and contribution to pork-related human cases despite causing little disease in pigs (Campos et al., 2019; Soliani et al., 2023).

Swine also host *S.* Choleraesuis, a pig-adapted serovar that historically caused severe septicemia and pneumonia with high mortality in young pigs. Although primarily adapted to swine, *S.* Choleraesuis remains clinically important due to its capacity to cause severe invasive disease outside its primary host; in rare cases, it can infect humans and cause invasive septicemic illness, highlighting that host-adapted serovars remain a zoonotic threat under the right conditions (Soliani et al., 2023). Its prevalence has declined in Western intensive systems but still persists in parts of Asia. Though rare in humans, *S.* Choleraesuis can cause invasive bloodstream infections and dissemination between pigs (Leekitcharoenphon et al., 2019).

### 6.4 Companion animals

Dogs and cats can carry NTS but are not primary reservoirs. They typically acquire infection through raw meat diets, contaminated treats, or prey (Ahmed et al., 2021). Surveys show that 20% of raw pet food products contain *Salmonella* in Canada, and pets fed raw diets shed the pathogen more often than those on cooked diets. Most infected pets remain asymptomatic, though they may shed bacteria for days or weeks (Davies et al., 2019).

However, the main concern is zoonotic transmission: outbreaks of human salmonellosis have been traced to contaminated pet foods such as dry kibble and pig-ear treats (Finley et al., 2006), as well as indirect household transmission through pets exposed to contaminated diets (Ahmed et al., 2021). While no serovar is unique to pets, common serovars such as *S.* Typhimurium, *S.* Infantis, and *S.* Newport are often isolated. Cat-associated cases linked to wild-bird predation (*S.* Typhimurium) highlight their role as incidental reservoirs bridging wildlife and humans (Ahmed et al., 2021).

### 6.5 Wildlife

Wildlife plays an important role in *Salmonella* ecology by sustaining reservoirs and spreading generalist serovars. Wild birds, especially passerines and pigeons, frequently shed *Salmonella* after environmental exposure and occasionally suffer mass die-offs from host-adapted *S.* Typhimurium variants (e.g., DT2 in pigeons) (Wigley, 2024a). While birds can pick up serovars from farms, they also contribute to recycling and dispersal in the environment. Human cases linked to backyard feeders highlight direct zoonotic spillover (Wigley, 2024a).

Reptiles and amphibians are natural carriers, with ∼30%–40% of known serovars associated with cold-blooded hosts. Turtles, lizards, and snakes shed *Salmonella* persistently without illness, and reptile exposure accounts for ∼3%–7% of human NTS infections in some regions (Pees et al., 2023). Mammalian wildlife such as wild boars, deer, raccoons, and rodents also harbor *Salmonella*, often reflecting farm contamination but enabling reintroduction to livestock, exemplifying the wildlife–livestock–human interface (Hilbert et al., 2012).

Animal reservoirs continually seed the environment and food chain, maintaining exposure pathways that link livestock, wildlife, and humans. Understanding how these exposures translate into long-term colonization and shedding in people is therefore essential for explaining NTS persistence in humans. Persistence within animal reservoirs not only sustains endemic circulation in herds and flocks, but also directly influences human persistence, as selection for antimicrobial resistance and stress-tolerant strains in livestock can prolong colonization and carriage following zoonotic transmission (Section “3 Persistence in humans”) and contribute to environmental amplification through manure and agricultural runoff (Section “7 Environmental persistence and transmission routes”).

## 7 Environmental persistence and transmission routes

Non-typhoidal *Salmonella* can survive for extended periods across agricultural environments, enabling long-term circulation among animals, soil, and crops. Biological vectors such as flies, rodents, and other wildlife further facilitate the movement of *Salmonella*, extending transmission pathways throughout the agricultural landscape (Wales et al., 2010). Persistence in food-processing environments is strongly associated with the ability of *Salmonella* to form robust biofilms on stainless steel and other industrial surfaces (Vestby et al., 2009). This shows that NTS persistence is maintained across multiple environments, complicating control efforts and underscoring the need for coordinated mitigation strategies across the entire food-production continuum.

### 7.1 Survival in soil and water environments

In soil, NTS can remain viable for months under favorable conditions. 14 *Salmonella* spp. survived up to 216 days in nutrient-rich loam soil, whereas survival was shorter in low-organic sandy soil (Alegbeleye and Sant’Ana, 2023). Temperature interacts with soil type to influence longevity: some *Salmonella* serovars persisted best at cooler temperatures (∼5 °C), while others survived longer at 30–37 °C (Alegbeleye and Sant’Ana, 2023). The presence of organic material, such as manure amendments, greatly enhances *Salmonella* survival in soils (Ramos et al., 2021). Field trials have documented NTS persisting for 5–7 months in manure-amended farm soils (Gu et al., 2019), highlighting how moisture and nutrients in organic matter help shield bacteria from environmental stress and promote long-term survival. In manure-amended soils, NTS has been detected months after application, with some studies reporting viability for up to 332 days (You et al., 2006). Field-scale crop–livestock systems likewise demonstrate that *Salmonella* can persist in soil and on produce well beyond typical pre-harvest intervals, indicating that environmental reservoirs on farms play a substantial role in sustaining contamination risks (Goodwyn et al., 2023). Recent studies further emphasize that environmental persistence of NTS is strongly influenced by interactions among temperature, moisture availability, biofilm formation, agricultural runoff, and organic nutrient content, which together shape survival dynamics in soil ecosystems and contribute to dissemination through manure-amended agricultural systems (Billah and Rahman, 2024; Woodford et al., 2024; Ranjan et al., 2026).

In aquatic environments, NTS can also persist for extended periods. Viability mechanisms (e.g., entry into biofilms or protozoa) enable *Salmonella* to survive for several months in natural waters (Liu et al., 2023). Under typical conditions of variable temperature, sunlight (UV exposure), and limited nutrients, however, *Salmonella* populations in water tend to dwindle within a few weeks, often less than 30 days (Liu et al., 2023). Still, biofilm formation in aquatic systems can facilitate extended viability of NTS in rivers, ponds, irrigation systems, and water distribution networks. Recent evidence further suggests that flooding events, climate-associated environmental changes, and microbial interactions within aquatic biofilms can enhance the persistence and spread of NTS in environmental waters (Kim et al., 2025; Sosah et al., 2025; Avcı et al., 2026; Ranjan et al., 2026). These findings show that environmental factors such as temperature, moisture levels, sunlight exposure, and organic nutrient availability critically determine how long NTS can persist in soil or water.

### 7.2 Persistence on farms and in food facilities

On animal farms, NTS frequently persists within specific environmental niches, including barns, pens, and pastures. For example, in poultry operations, *Salmonella* can survive between production cycles by colonizing litter, dust, or equipment, leading to infection of new flocks even in the absence of vertical transmission (Pang et al., 2023). Areas with elevated moisture, such as manure piles, water troughs, or damp bedding, can support *Salmonella* survival on farms, while rodents and insects may further promote within-farm dissemination. Effective sanitation, pest control, and waste-management practices are therefore critical to disrupt these environmental reservoirs on farms (Andino and Hanning, 2015; Meerburg and Kijlstra, 2007).

In food-processing and packing facilities, *Salmonella* demonstrates a remarkable capacity for persistence, largely through the formation of resilient biofilms on food-contact and industrial surfaces. Biofilm-forming serovars have been repeatedly recovered from processing environments, particularly within poultry and meat production lines (Pang et al., 2023). Notably, NTS strains repeatedly isolated from feed and fish-meal factories exhibit enhanced biofilm-forming capacity, enabling survival through sanitation cycles and contributing to recurrent contamination events (Vestby et al., 2009).

*Salmonella* biofilms on stainless steel, plastic, and other food-contact surfaces increase bacterial viability and protect cells from cleaning and disinfection, thereby contributing to persistent contamination and recurring cross-contamination of products (Ivers et al., 2024; Joseph et al., 2001). These biofilm communities can withstand routine disinfection; studies show that mixed-species biofilms containing *Salmonella* often survive standard sanitizer treatments and can even grow in hard-to-reach niches after cleaning (Ban-Cucerzan et al., 2025; Wang Y. et al., 2025). Moreover, *Salmonella* exhibits pronounced tolerance to desiccation. Under dry conditions, bacteria can enter dormant, stress-resistant states, allowing prolonged survival on equipment or packaging surfaces, particularly in moderately humid areas such as refrigerated storage rooms where complete drying is avoided (Pang et al., 2023). In low-moisture foods, including powdered milk, nuts, cereals, and pet food, *Salmonella* can survive for months to years owing to its high tolerance to desiccation and heat (Beuchat et al., 2013). These adaptations allow persistent environmental contamination and create opportunities for re-introduction into the food chain.

Beyond phenotypic resistance patterns, NTS carries antimicrobial-resistance traits that further promote long-term persistence across environmental and food-production settings. Resistance to key drug classes, including fluoroquinolones and β-lactams, enhances bacterial survival under antimicrobial and oxidative stress and is closely associated with tolerance mechanisms that facilitate persistence within biofilms and other environmental niches (Hill et al., 2021; González et al., 2018). At farm and processing levels, multidrug-resistant strains are frequently maintained under antimicrobial and sanitation pressures, allowing resistant lineages to persist within poultry production systems and food-processing environments (Hetman et al., 2022).

Importantly, antimicrobial-resistance traits often co-occur with stress-response and biofilm-associated phenotypes that protect *Salmonella* from disinfectants, desiccation, and nutrient limitation. Biofilm-forming, multidrug-resistant strains exhibit enhanced persistence on abiotic surfaces, such as stainless steel and plastic, enabling survival through routine cleaning and facilitating recurrent contamination events (Vestby et al., 2009; Ban-Cucerzan et al., 2025). Environmental surveillance further indicates that resistance determinants are commonly associated with mobile genetic elements that circulate among animal, human, and environmental compartments, reinforcing persistence and dissemination across One Health systems (Hendriksen et al., 2019; Hetman et al., 2022). Recent reviews emphasize that antimicrobial resistance should therefore be regarded not only as a therapeutic challenge but also as a persistence-enabling trait that stabilizes *Salmonella* populations under selective pressure (Lamichhane et al., 2024; Gomes et al., 2025).

Collectively, these findings underscore that environmental persistence and endurance of NTS bridge animal, human, and ecological domains. Soil, water, vectors, and industrial surfaces form interconnected reservoirs that sustain transmission over extended time scales. Environmental reservoirs therefore function as both sinks and sources for *Salmonella*, enabling re-introduction into animal hosts and subsequent zoonotic transmission to humans, particularly in systems where contaminated soils, surface waters, or food-processing environments interface directly with livestock production (Sections 6 and 3). As illustrated in Figure 1, antimicrobial resistance selection interacts with environmental survival and host-associated carriage to reinforce persistence cycles across the human–animal–environment interface.

## 8 Serovar-specific mechanisms of persistence

Persistence of NTS is not governed by a single conserved mechanism but instead reflects a diversity of strategies that vary across serovars, host species, and environmental contexts (Ehrhardt et al., 2023; Cheng et al., 2019). These strategies are shaped by differences in host adaptation, ecological niches, transmission pathways, and exposure to antimicrobial and environmental stressors (Tack et al., 2020). As a result, persistence must be understood as a serovar- dependent and context- specific phenomenon rather than a uniform biological trait.

To provide a comparative synthesis, Table 1 summarizes key persistence mechanisms across major NTS serovars, highlighting variation in dominant reservoirs, ecological settings, and underlying biological strategies. Broadly, these mechanisms can be categorized into:

**TABLE 1:** Serovar-specific persistence mechanisms of non-typhoidal *Salmonella*.

Serovar	Primary reservoirs	Dominant persistence mechanisms	Key ecological/clinical settings	One Health relevance
*Salmonella* Typhimurium	Humans, livestock, wildlife	Intracellular survival in macrophages; immune modulation; metabolic and stress adaptation (PhoP/PhoQ, RpoS); prolonged intestinal carriage; biofilm-like aggregation (Monack et al., 2004; Lawley et al., 2006; Kingsley et al., 2000, 2002; Ehrhardt et al., 2023)	Human hosts; animal reservoirs; lymphatic tissues; intestinal tract	Sustains asymptomatic carriage and intermittent shedding, enabling re-seeding of animal and environmental reservoirs (Marzel et al., 2016; Waldner et al., 2012)
*Salmonella* Heidelberg	Poultry; farm and processing environments	Environmental survival; robust biofilm formation; desiccation tolerance; multidrug resistance; sanitizer tolerance (Vestby et al., 2009; Webber et al., 2019; Etter et al., 2019; Melo et al., 2021)	Poultry farms; litter; processing facilities; food-contact surfaces	Links agro-environmental persistence with foodborne outbreaks and recurrent human exposure (Webber et al., 2019; Danasekaran, 2024; Desvars-Larrive et al., 2024)
*Salmonella* Enteritidis	Poultry	Reproductive tract colonization; vertical transmission; asymptomatic carriage in adult hens (Chen et al., 2021; Wigley, 2024b)	Eggs; poultry production systems	Enables silent persistence and sustained human exposure via egg-associated transmission (Shaji et al., 2023; Wigley, 2024b)
*Salmonella* Dublin	Cattle	Host adaptation; long-term carriage; intermittent shedding; invasive disease potential (Nielsen, 2013; Holschbach and Peek, 2018)	Dairy herds; milk; farm environments	Maintains endemic circulation in cattle with high zoonotic risk and severe human disease (Velasquez-Munoz et al., 2024)
*Salmonella* Newport	Livestock; environment	Environmental persistence; survival in manure-amended soils; antimicrobial resistance (You et al., 2006; Ramos et al., 2021)	Farms; produce systems; soil and water	Bridges agricultural environments and human exposure pathways, particularly via fresh produce (Kovac et al., 2017; Goodwyn et al., 2023)
Monophasic *S.* Typhimurium (4,[5],12:i:-)	Swine	Efficient herd-level transmission; prolonged shedding; high antimicrobial resistance (Mastrorilli et al., 2018; Campos et al., 2019; Soliani et al., 2023)	Pig production systems	Amplifies persistence in swine and contributes to resistant zoonotic transmission (Soliani et al., 2023)

Major non-typhoidal *Salmonella* serovars differ in their dominant reservoirs and persistence strategies, including host-associated adaptation, environmental endurance, biofilm formation, and antimicrobial resistance. Together, these mechanisms illustrate how persistence across human, animal, and environmental compartments sustains transmission within a One Health framework.

(i) host-associated persistence (e.g., intracellular survival and chronic carriage),

(ii) environmental persistence (e.g., biofilm formation, desiccation tolerance, and survival in soil and water). (iii) antimicrobial resistance–associated persistence, which enhances survival under selective pressures across both host and environmental compartments (Tack et al., 2020; Vestby et al., 2009; Gomes et al., 2025).

Generalist serovars such as *S.* Typhimurium and *S.* Enteritidis demonstrate high adaptability across multiple reservoirs, enabling persistence through combined host-associated and environmental mechanisms (Cheng et al., 2019; Soliani et al., 2023). In contrast, host-adapted serovars such as *S.* Dublin rely primarily on long-term colonization within specific animal populations, often with intermittent shedding that sustains endemic transmission (Nielsen, 2013; Holschbach and Peek, 2018). Other serovars, including *S.* Newport and *S.* Heidelberg, exhibit strong environmental persistence linked to survival in agro-ecosystems, biofilm formation, and resistance to sanitation procedures, facilitating repeated contamination of food systems (Vestby et al., 2009; Webber et al., 2019). Monophasic variants of *S.* Typhimurium further highlight how genomic evolution and antimicrobial resistance can enhance persistence and dissemination within intensive production systems (Mastrorilli et al., 2018; Campos et al., 2019).

While these patterns illustrate broad trends, they should be interpreted as part of a continuum, where many serovars combine multiple persistence strategies depending on ecological context. Importantly, persistence in one domain (e.g., livestock or environment) often drives persistence in others through continuous reseeding across the human–animal–environment interface (Velasquez-Munoz et al., 2024; Soliani et al., 2023)

To illustrate these mechanisms in greater detail, selected serovars are discussed below as representative examples, recognizing that these do not encompass the full diversity of NTS persistence strategies.

### 8.1 Representative host-associated persistence: *Salmonella* Typhimurium

*Salmonella* Typhimurium is one of the most extensively studied NTS serovars and has provided valuable insight into host-associated persistence mechanisms. Its ability to persist within hosts is linked to intracellular survival within macrophages and epithelial cells, modulation of host immune responses, and prolonged intestinal colonization with intermittent shedding (Monack et al., 2004; Lawley et al., 2006).

Experimental studies indicate that persistence is supported by coordinated stress-response pathways, metabolic adaptation, and immune modulation, allowing bacterial survival under nutrient limitation, oxidative stress, and antimicrobial pressure (Kurtz et al., 2020; Desai et al., 2023). These features contribute to asymptomatic carriage and sustained fecal shedding in both humans and animal hosts.

However, it is important to note that much of the mechanistic understanding of *S.* Typhimurium persistence derives from animal models, particularly murine systems that primarily reflect systemic infection dynamics and tissue culture models. While these models provide valuable insight into intracellular survival and immune evasion, they may not fully represent intestinal persistence and transmission observed in typical human NTS infections (Ehrhardt et al., 2023). Therefore, findings from these models should be interpreted within the broader ecological and serovar-specific context summarized in Table 1.

### 8.2 Representative environmental persistence: *Salmonella* Heidelberg

In contrast to host-associated persistence, *S.* Heidelberg exemplifies a persistence strategy strongly linked to environmental survival within agro-ecosystems and food-production environments. This serovar is frequently associated with poultry production systems, where it can persist in litter, dust, water systems, and processing equipment (Voss-Rech et al., 2019).

A defining feature of *S.* Heidelberg is its capacity for robust biofilm formation on abiotic surfaces, including stainless steel and plastic, which enhances survival under cleaning and disinfection procedures (Vestby et al., 2009; Melo et al., 2021). This capability is often coupled with tolerance to environmental stressors such as heat, desiccation, and exposure to sanitizers, allowing persistence across production cycles and facilitating repeated contamination of food products (Etter et al., 2019).

Antimicrobial resistance further contributes to persistence by enhancing tolerance to both antimicrobial agents and environmental stress conditions. Multidrug-resistant *S.* Heidelberg strains have been repeatedly associated with poultry-related outbreaks, indicating that resistance traits may function not only as clinical challenges but also as persistence-enabling adaptations within agro-environmental systems (Melo et al., 2021; Public Health Agency of Canada [PHAC], 2025b).

### 8.3 Implications for One Health persistence

Together, these examples illustrate that persistence strategies differ markedly across serovars but are unified by their contribution to sustained transmission within interconnected systems. Host-associated persistence promotes long-term carriage and shedding, while environmental persistence enables survival outside the host and repeated reintroduction into the food chain. Antimicrobial resistance acts as a cross-cutting factor that enhances survival and stability under selective pressures across all compartments (Gomes et al., 2025).

From a One Health perspective, these findings emphasize that effective mitigation requires integrated strategies targeting multiple persistence pathways simultaneously, rather than focusing on a single host or environment. As summarized in Table 1, linking serovar-specific biology with ecological context is essential for understanding and disrupting persistence cycles across the human–animal–environment interface.

## 9 Strategies to mitigate NTS infections: persistence and One Health approach

As illustrated in Figure 1, the persistence of NTS across interconnected human, animal, and environmental compartments necessitates integrated mitigation strategies that target multiple reservoirs and transmission pathways within a One Health framework.

### 9.1 Biological interventions

Biological interventions targeting *Salmonella* increasingly aim not only to reduce acute infection but also to limit long-term persistence, environmental shedding, and transmission across the One Health continuum.

Vaccination remains a vital strategy for controlling *Salmonella* in animal reservoirs. In poultry, several vaccine platforms, including live-attenuated, inactivated, and recombinant types, have been implemented to induce strong immune protection and reduce *Salmonella* colonization. These programs have substantially lowered flock infection rates over recent decades, improving food safety outcomes (Pan et al., 2024). Vaccination can decrease *Salmonella* prevalence in flocks, with significant reductions in fecal shedding and egg contamination (Dórea et al., 2010; Huberman et al., 2022). Live-attenuated vaccines such as Vaxsafe STM1 or recombinant subunit formulations stimulate strong mucosal and systemic immunity and help maintain gut microbial balance during challenge. However, their long-term efficacy may vary across *Salmonella* serovars and production environments, highlighting the need for continuous optimization and DIVA (Differentiating Infected from Vaccinated individuals)-compatible designs (McWhorter and Chousalkar, 2018). Importantly, while vaccination reduces disease burden and shedding, it does not fully eliminate *Salmonella* persistence, and vaccine performance may be influenced by gut microbiome composition and environmental exposure.

From a One Health perspective, this vaccination approach aligns closely with integrated control strategies: by targeting *Salmonella* at its animal source, the risk of human exposure through food is reduced. While humans can be vaccinated against typhoidal *Salmonella* Typhi, for non-typhoidal serovars, the emphasis remains on preventing infection in livestock and food chains (Nazir et al., 2025).

Bacteriophage therapy offers a targeted alternative to antibiotics through phages that infect and lyse *Salmonella*. More than forty phage-based products are currently marketed worldwide for food-sector applications aimed at reducing contamination and zoonotic transmission (Segundo-Arizmendi et al., 2025). Field-scale implementation has been reported, including INSPEKTOR^®^ use in Brazilian poultry systems with reductions of 0.5–6 logs (Pino et al., 2025), while controlled studies demonstrate significant reductions in intestinal burdens in pigs and poultry (Wall et al., 2010). However, adoption of phage therapy remains constrained by manufacturing challenges, limited regulatory standardization, and the risk of resistance evolution (Segundo-Arizmendi et al., 2025; Gildea et al., 2022). Despite these limitations, phage therapy remains attractive due to specificity, microbiome preservation, and adaptability through cocktail design, with ongoing work focused on improving delivery and integration into food safety frameworks (Strathdee et al., 2023; Segundo-Arizmendi et al., 2025).

In both humans and animals, probiotics and prebiotics have been widely explored to reduce Salmonella colonization and fecal shedding. Probiotic strains (e.g., *Lacticaseibacillus rhamnosus* GG, *Bifidobacterium* spp.) and engineered microbes (e.g., *E. coli* Nissle 1917) can competitively exclude *Salmonella* and modulate host immunity, with strong reductions reported in poultry models (Forkus et al., 2017; Closs et al., 2025). Prebiotics such as mannan-oligosaccharides (MOS) and fructo-oligosaccharides (FOS) may reduce adhesion and support short-chain fatty acids (SCFAs) production (Alsherify and Hassanabadi, 2024; Malli et al., 2025; Singh et al., 2025). These approaches may be further enhanced through symbiotic strategies and supplementation alongside vaccination to reduce shedding and transmission (El-Shall et al., 2020; Shehata et al., 2022). Precision microbiome engineering is emerging as a strategy to limit *Salmonella* persistence and AMR dissemination through engineered probiotics delivering antimicrobial peptides or CRISPR–Cas systems to selectively target pathogens or cure resistance plasmids, as well as quorum-sensing disruption to reduce virulence and biofilm-associated persistence (Liu et al., 2024; Navalkele and Chopra, 2024). However, these advanced approaches remain early-stage and require further validation of safety, stability, and feasibility before widespread implementation.

### 9.2 Environmental and farm-level controls

Because *Salmonella* can persist in farm environments for extended periods, strict biosecurity and hygiene measures are essential to limit its introduction, amplification, and dissemination. These interventions are specifically designed to disrupt the persistence mechanisms described in Section “7 Environmental persistence and transmission routes,” including biofilm formation, desiccation tolerance, and survival in organic farm residues. In practice, the pathogen is frequently introduced and maintained through rodents, wild birds, insects, contaminated equipment, and human traffic; therefore, integrated pest control programs, including rodent trapping, bird-proofing, insect management, and wildlife exclusion represent critical components of effective biosecurity (Gentile et al., 2025). Routine cleaning and disinfection of animal housing, equipment, and transport vehicles, combined with covered feed and water systems and appropriate manure handling, further reduce environmental reservoirs. Consistent with this, a large-scale meta-analysis confirmed that layered biosecurity strategies, integrating cleaning and disinfection, feed and water control, and pest exclusion, are associated with substantially lower *Salmonella* prevalence on farms (Pedersen et al., 2025).

From a One Health perspective, controlling *Salmonella* requires intervention at every stage of the “farm-to-fork” continuum. Beyond primary production, strict sanitation and hygiene during animal transport, slaughter, and processing are essential to prevent cross-contamination. For example, implementation of rigorous hygiene protocols in slaughterhouses, regular equipment disinfection, and adequate thermal processing have been shown to significantly reduce contamination risks in food products (Betiku et al., 2025). At the population level, public health interventions, including consumer education on safe food handling, proper refrigeration, and hand hygiene after animal contact, further reduce human exposure (Ehuwa et al., 2021). The value of coordinated regulatory action is illustrated by Canadian interventions targeting frozen breaded chicken products, where mandatory pre-cooking prior to retail sale reduced *Salmonella* prevalence from 28% to 2.9% and was associated with a 23% reduction in reported human cases (Kanoatova et al., 2024). Collectively, these findings demonstrate that environmental control and biosecurity are foundational pillars that complement biological interventions, reinforcing protection of human health across the One Health interface.

Litter management represents a critical component of farm-level *Salmonella* control, particularly in poultry and other intensive production systems where bedding can act as a long-term environmental reservoir. Studies show that *Salmonella* can persist for weeks to months in poultry litter, and survival is strongly influenced by moisture, organic load, pH, and temperature, mirroring the environmental persistence traits outlined in Section “7 Environmental persistence and transmission routes” (Wilkinson et al., 2011; Avidov et al., 2021; Gutierrez and Schneider, 2022). This issue is especially relevant because poultry litter is commonly reused as fertilizer, creating potential pathways for dissemination into produce systems if treatment is inadequate (Gutierrez and Schneider, 2022). Extended downtime, litter aging, and windrowing/composting approaches promote pathogen die-off through thermophilic activity and microbial competition, leading to substantial reductions in *Salmonella* viability (Wilkinson et al., 2011; Avidov et al., 2021). Chemical amendments, including acidifiers and alkalizing agents, further suppress survival by altering pH and water activity while improving litter quality and reducing ammonia emissions (Line, 2002; Bennett et al., 2003; Pineda et al., 2021; Gutierrez and Schneider, 2022). Manure removal, composting, and controlled storage also reduce opportunities for amplification and dissemination into soil and water reservoirs (Hutchison et al., 2005; Chen et al., 2014). Finally, worker hygiene and movement control remain essential, as contaminated hands, footwear, clothing, and equipment can facilitate within-farm transmission, and measures such as hand washing, dedicated clothing, and footbath stations reduce cross-contamination (Huneau-Salaün et al., 2009; Delpont et al., 2021).

Beyond conventional cleaning and disinfection, bioremediation and phytoremediation offer sustainable strategies to mitigate *Salmonella* maintenance in the environment. These approaches also aim to counteract long- term environmental survival mechanisms described in Section “7 Environmental persistence and transmission routes.” Bioremediation employs beneficial microbial consortia or engineered bacteria to degrade organic residues and suppress *Salmonella* in soil, manure, and wastewater systems (Singh et al., 2020; Sharma et al., 2023). Phytoremediation leverages plant–microbe interactions in buffer strips and wetlands to filter contaminated runoff. In these systems, plants such as *Typha* spp. and *Phragmites australis* support antagonistic rhizosphere bacteria that can trap and reduce *Salmonella* (Zhang et al., 2025). When integrated into manure and waste management practices, these solutions lower pathogen loads entering surrounding ecosystems. Despite their potential, these approaches remain context-dependent and require further validation at scale under commercial farming conditions, particularly with respect to long-term efficacy, seasonal variability, and regulatory implementation.

### 9.3 Surveillance and detection approaches

Detection and characterization of *Salmonella* across human, animal, and environmental systems remain essential components of surveillance and persistence control. Current approaches combine culture-based methods, which enable confirmation and antimicrobial susceptibility testing, with molecular tools that provide rapid and sensitive detection. These complementary strategies support timely identification of contamination sources and facilitate intervention across the One Health continuum.

qPCR is widely used for rapid detection of *Salmonella* in clinical, food, and environmental samples, supporting early identification of contamination and surveillance activities (Fröder et al., 2025; Isaac et al., 2025; Wang H. et al., 2025). Advances such as multiplex assays, portable platforms, and viability-based approaches, e.g., “propidium monoazide (PMA)-qPCR” have improved field applicability and reduced overestimation by distinguishing live from non-viable cells in complex matrices such as wastewater and manure (von Hertwig et al., 2024).

Whole-genome sequencing (WGS) has become the gold standard for high-resolution characterization of *Salmonella*, enabling strain-level identification and detailed analysis of antimicrobial resistance, plasmids, and host adaptation (Ferrer-Bustins et al., 2024; Turcotte et al., 2022). These approaches provide insight into persistence-associated traits, including stress survival and plasmid-mediated resistance, and support transmission tracking across human, animal, and environmental reservoirs (Gomes et al., 2025; Wilson et al., 2025). Genomic surveillance has also highlighted the dissemination of resistant lineages, including those associated with mobile genetic elements across animal populations (Alba et al., 2020; Li et al., 2024; Papić et al., 2022; de Almeida Figueira et al., 2025; Hetman et al., 2022; Núncio et al., 2022). Shotgun metagenomics is increasingly used to detect *Salmonella*, profile resistomes, and infer genetic exchange without the need for culture (Quek et al., 2025; Shang et al., 2025).

Integrated workflows that combine molecular screening, culture confirmation, and genomic analysis provide a balanced and effective approach to surveillance (Malorny et al., 2004). While molecular assays alone may overestimate prevalence due to detection of non-viable DNA, and culture methods may underestimate diversity, combining both improves sensitivity and specificity and enhances outbreak investigation and persistence tracking (Ríos-Castillo et al., 2022; Turcotte et al., 2022).

Finally, standardization of detection methods and reporting frameworks remains essential for effective One Health surveillance. Harmonized approaches, such as coordinated genomic reporting systems, facilitate data sharing and comparability across human, animal, food, and environmental sectors (European Food Safety Authority [EFSA] et al., 2022; Davedow et al., 2022). Continued integration of sequencing pipelines with surveillance systems is increasingly important for improving antimicrobial targeting, supporting decision-making, and strengthening responses to persistent *Salmonella* transmission (Quek et al., 2025; Gomes et al., 2025).

### 9.4 AI and data- driven approaches

Artificial intelligence (AI) and digital technologies are increasingly being incorporated into *Salmonella* surveillance frameworks to enhance early detection and support risk assessment. Machine-learning models integrating meteorological, farm-management, and genomic data can predict outbreak risk and identify hotspots before clinical detection (Garcia-Vozmediano et al., 2024), and AI-driven platforms combining farm, environmental, and veterinary data streams have shown utility in early-warning and on-farm monitoring systems (Garcia-Vozmediano et al., 2024; Pillai et al., 2022; Ram Das et al., 2024). Digital traceability tools, including blockchain-based systems and digital-twin models linked to Internet-of-Things (IoT) sensors, are also being applied across the food supply chain to improve transparency and traceback capacity by monitoring animal movement and production conditions (Ellahi et al., 2024; Huang et al., 2024a; Roumeliotis et al., 2024). When coupled with genomic surveillance, these approaches support faster source identification and more data-informed interventions, strengthening coordinated One Health prevention efforts. Emerging data- driven approaches, including machine- learning models and integrated genomic surveillance platforms, are expected to enhance predictive capacity and support targeted interventions across One Health systems.

### 9.5 Experimental frameworks to investigate serovar-specific persistence

Although *S.* Typhimurium has provided foundational insight into host-associated persistence, evidence indicates that persistence strategies vary substantially across non-typhoidal *Salmonella* serovars, with strains exhibiting distinct survival profiles across environmental matrices, animal reservoirs, and host-associated niches that reflect adaptation to specific ecological pressures and transmission routes (Ehrhardt et al., 2023). Accordingly, future studies should adopt comparative experimental frameworks that assess strain- and serovar-level persistence under controlled environmental conditions (e.g., soil, water, manure, and food-contact surfaces), alongside *in vivo* colonization and shedding dynamics in relevant animal hosts, to evaluate how specific strains tolerate stress and establish long-term reservoirs across the human–animal–environment interface. Coupling these models with comparative bioinformatics, including whole-genome sequencing and pan-genome analyses, can identify persistence determinants beyond classical virulence factors by linking phenotypes to stress-response pathways, biofilm-associated genes, metabolic adaptations, and plasmid-borne traits, including antimicrobial resistance (Gomes et al., 2025). Integrating longitudinal *in vivo* data with genome-wide association analyses and functional validation can further distinguish conserved mechanisms from serovar-specific adaptations, supporting predictive One Health strategies to mitigate *Salmonella* persistence.

## 10 Conclusion

Persistence of non-typhoidal *Salmonella* is a multifactorial challenge shaped by bacterial adaptation, host susceptibility, and environmental resilience. Mechanisms, including intracellular survival, biofilm formation, stress tolerance, metabolic flexibility, and antimicrobial resistance genes, enable *Salmonella* to persist across human, animal, and environmental reservoirs and to re-enter transmission cycles. Importantly, persistence is not uniform across serovars. While *S.* Typhimurium has served as a key model, growing evidence indicates that other clinically relevant serovars rely on distinct host-associated and agro-environmental survival strategies. Serovars such as *S.* Heidelberg, along with other emerging lineages, remain comparatively understudied and warrant deeper investigation to better understand their mechanisms of persistence. From a One Health perspective, effective mitigation therefore requires coordinated interventions across sectors, including vaccination, probiotics, bacteriophage therapy, and environmental controls targeting litter, manure, and farm infrastructure. Advances in molecular diagnostics, whole-genome sequencing, and AI-assisted surveillance further strengthen early detection, traceback, and targeted control. However, critical gaps remain in understanding long-term human carriage, environmental re-infection, and the ecological role of antimicrobial resistance in sustaining persistence. Addressing these gaps will require integrated surveillance and comparative multi-strain approaches supported by persistence assays and longitudinal sampling across human, animal, and environmental sources.
